# Induction of a tumor-metastasis-receptive microenvironment as an unwanted and underestimated side effect of treatment by chemotherapy or radiotherapy

**DOI:** 10.1186/1757-2215-6-95

**Published:** 2013-12-27

**Authors:** Mariusz Z Ratajczak, Tomasz Jadczyk, Gabriela Schneider, Sham S Kakar, Magda Kucia

**Affiliations:** 1 Stem Cell Institute at the James Graham Brown Cancer Center, University of Louisville, 500 S. Floyd Street, Rm. 107, Louisville, KY 40202, USA; 2 Third Division of Cardiology, Silesian Medical University, Katowice, Poland

**Keywords:** Cancer metastasis, Side effects, Chemotherapy, Radiotherapy, SDF-1, CXCR4, S1P, C1P, Alarmines

## Abstract

There are well-known side effects of chemotherapy and radiotherapy that are mainly related to the toxicity and impaired function of vital organs; however, the induction by these therapies of expression of several pro-metastatic factors in various tissues and organs that *in toto* create a pro-metastatic microenvironment is still, surprisingly, not widely acknowledged. In this review, we support the novel concept that toxic damage in various organs leads to upregulation in “bystander” tissues of several factors such as chemokines, growth factors, alarmines, and bioactive phosphosphingolipids, which attract circulating normal stem cells for regeneration but unfortunately also provide chemotactic signals to cancer cells that survived the initial treatment. We propose that this mechanism plays an important role in the metastasis of cancer cells to organs such as bones, lungs, and liver, which are highly susceptible to chemotherapeutic agents as well as ionizing irradiation. This problem indicates the need to develop efficient anti-metastatic drugs that will work in combination with, or follow, standard therapies in order to prevent the possibility of therapy-induced spread of tumor cells.

## Introduction

The results of conditioning strategies for bone marrow (BM) transplantation by employing myelo- or submyeloablative doses of chemotherapy or radiotherapy provides evidence that damage of the BM environment results in upregulation of several factors that direct chemottraction and homing of hematopoietic stem progenitor cells (HSPCs) to BM. The most important homing signal responsible for this effect is mediated by α-chemokine stromal-derived factor 1 (SDF-1) [1,2]. The biological effect of this chemokine is supported by other factors, including bioactive phosphosphingolipids such as sphingosine-1-phosphate (S1P) and ceramide-1-phosphate (C1P) [3-7], as well as some members of a family of alarmines, including extracellular nucleotides such as ATP and UTP [8,9], that are released from damaged and “leaky” BM cells. All these factors direct migration and translocation of HSPCs circulating in peripheral blood (PB) into BM niches. Moreover, the same factors are known to be upregulated in organs damaged by hypoxia, as seen in acute myocardial infarction, stroke, ischemic kidney damage, or toxic liver injury, which chemoattract normal circulating stem cells for potential tissue/organ regeneration [10-14].

At the same time, all these factors are very well-known chemoattractants for several types of malignant cells and are involved in formation of distant metastases [15-20]. It is well known that metastases are responsible for more than 90% of tumor-associated mortality and thus are a crucial problem for developing more efficient and successful cancer therapies. Depending on the tumor type and microenvironment, as postulated 125 years ago by Paget in a famous “seed and soil hypothesis” [21,22], tumor cells have a preference for metastasizing to “tumor-receptive” organs. This receptiveness depends on a repertoire of chemoattractants secreted in a given tissue as well as expression of corresponding receptors on the surface of the circulating/migrating tumor cells. An important role is also played by tumor cell-expressed adhesion molecules that anchor circulating tumor cells to the endothelium at the site of the distant metastasis [23,24].

One of the most important questions in oncology remains the true nature of the tumor cells that are endowed with migratory and thus metastatic properties. It is obvious that most growing tumors are composed of malignant cells that show different levels of maturation. Usually in a growing solid tumor, the more differentiated cells are seen in its central areas, and more primitive cells endowed with migratory/infiltration properties are encountered on the periphery [25].

According to accumulating evidence, the rarest tumor cells are cancer stem cells (CSCs), which are more resistant to treatment by standard therapies and, like most normal stem cells during embryogenesis, are strongly endowed with migratory properties [26-32]. Another possibility that has been postulated as a source of metastatic cancer cells is the epithelial-to-mesenchymal transition (EMT), in which some tumor cells, by changing their genetic program, become highly migratory cells [30,33-35]. Thus, as is currently speculated, both of these processes - the presence of CSCs in the growing tumor as well EMT - may drive tumor metastasis and are responsible for tumor dissemination to distant organs.

### Lessons from normal circulating stem cells during tissue/organ injury

One of the important features of early-development stem cells is their propensity to migrate, which is clearly seen for many types of stem cells during processes such as gastrulation and subsequent organ development [36-39]. Later on in adult life, some types of stem cells are still highly migratory and are mobilized into peripheral blood (PB) and lymph, migrating to distant locations [40,41]. The best examples for such stem cells are hematopoietic stem progenitor cells (HSPCs) [36,42], endothelial progenitor cells (EPCs) [43], mesenchymal stromal cells (MSCs) [44], and very small embryonic-like stem cells (VSELs) [45].

It is well known that all of these types of stem/progenitor cells are detectable under steady-state conditions in PB and lymph, and their number increases, for example, during organ damage or inflammation [46]. In all these situations, damaged tissues release potent chemotactic factors that direct trafficking of these cells. These pro-chemotactic compounds belong to families of chemokines, growth factors, bioactive lipids, and some small molecules known as alarmines [7-9,17]. Chemokines and some pro-angiopoietic growth factors are released from the damaged tissues in response to damage-related hypoxia involving hypoxia-inducible factor 1 alpha (HIF-1α) [47] activation of transcription of genes encoding chemokines (e.g., SDF-1; interleukin 8, IL-8) [48-51] and selected growth factors (e.g., vascular endothelial growth factor, VEGF) that are primarily involved in promoting vascularization of damaged organs.

Also released from damaged cells during tissue/organ injury are so-called alarmines or damage-associated molecular pattern molecules (DAMPs, also known as danger-associated molecular pattern molecules), which can initiate and perpetuate immune responses and subsequent repair processes [52,53]. The most important molecules from this family of mediators are purine metabolites, including nucleotides (e.g., ATP and UTP) [54,55], the chromatin-associated protein high-mobility group box 1 (HMGB1) [56-58], and S100 molecules, which belong to a multigenic family of calcium-modulated proteins [59-61]. All these molecules are released from damaged cells and, after they have reached the extracellular space, serve as “danger” signals. Finally, accumulating evidence suggests a role for bioactive phosphosphingolipids such as S1P and C1P in regulating trafficking of stem/progenitor cells. As we recently demonstrated, both S1P and C1P direct migration of HSPCs, MSCs, EPCs, and VSELs [7].

It is very likely that a proper repertoire of these factors upregulated in damaged tissues is important for mobilization into PB and subsequent homing to the damaged organs of specific subsets of circulating stem progenitor cells [62]. In support of this possibility, VEGF more selectively mobilizes EPCs into PB [63].

Release of normal stem cells into circulation and their subsequent homing into damaged tissues may play some role in regeneration of organs damaged by chemotherapy or radiotherapy. The beneficial effects of stem cells in regeneration of damaged tissues are mainly based on the paracrine effects of circulating stem cells [64-66] as well as some more direct effects such as for example their contribution to vasculogenesis [67].

### Circulating tumor cells

Unfortunately, it is well known that, like normal stem cells, cancer cells also circulate in PB and lymph and may be seeded to distant tissues. Evidence has even accumulated that these circulating cancer cells may be already present at locations distant from the tumor at very early stages of tumor development [68]. As a population of de-differentiated and transformed normal cells, the propensity of tumor cells to migrate most likely reflects the migratory nature of normal early-development stem cells. This propensity may be particularly important for the population of most-primitive cancer cells present in the growing tumor known as CSCs [26,27,29,32,69] or cancer cells endowed with migratory properties characteristic of mesenchymal cells generated in the process of EMT, which in consequence leads to formation of disseminating tumor cells (DTCs) [30,31].

Moreover, it is logical that circulating CSCs or DTCs respond to a similar repertoire of chemoattractants as normal cells circulating in PB. Extravasation of malignant cells to the sites of metastasis depends also on expression on their surface of adhesion molecules that may tether them to endothelium as well as expression of proteolytic enzymes such as various metalloproteinases (MMPs) that facilitate their migration and infiltration in the tissues [24,70,71]. Some evidence has accumulated that a metastasis-permissive microenvironment at a future site of metastasis has already been created some time before “arrival” of new unwanted inhabitants, CSCs or DTCs [61,72-74]. Moreover, it has even been also postulated that, in some cases, BM-derived cells are involved in induction of this pro-metastatic microenvironment at distant locations [61,72].

Therefore, despite significant reduction of tumor mass with initial treatment, some cancer cells more resistant to radiotherapy and chemotherapy (e.g., CSCs) may survive and after treatment respond to chemotactic cues induced by therapy at distant locations. It is well known that more primitive cells have a higher expression of drug efflux pumps [75-77], possess more efficient DNA-repair mechanisms [78,79], and are more resistant to hypoxia [47].

### Chemotherapy and radiotherapy create a pro-metastatic microenvironment in bone marrow and other tissues

Besides surgical removal of tumor tissue, chemotherapy and radiotherapy are the most important and efficient treatment modalities employed to treat therapy-susceptible malignancies. The main aim of treatment - to destroy tumor cells - is unfortunately usually associated with toxicity to non-tumor cells and different degrees of tissue and organ damage. The organ most sensitive to toxic damage by chemotherapeutics is usually BM. Toxic damage to BM may somewhat mimic the situation encountered during conditioning before hematopoietic transplantation, when BM-residing cells are severely damaged and, in parallel, several chemoattractants are released in the BM microenvironment that play a role in chemoattraction and homing of the infused in the graft normal HSPCs [1,2,46,80]. We postulate that in a similar way, these factors may chemottract tumor cells that survived initial treatment.

In support of this notion, as already demonstrated by us and other groups, exposure of mice to irradiation [81], cyclophosphamide [80], or vincristine [17,82] upregulates the levels of several chemokines and growth factors, such as SDF-1, HGF/SF, VEGF, and monocyte chemotactic protein-1 (MCP-1), in the BM microenvironment. In addition, as we have recently demonstrated, *in vivo* exposure of mice to irradiation and cyclophosphamide elevates BM expression of S1P and C1P, which are endowed with strong chemotactic properties against normal as well as malignant cells [7,17]. Exposure of BM to irradiation, similar as to chemotherapeutics, leads to release of several alarmines (e.g., ATP and UTP) from the damaged BM cells (manuscript in preparation). We can assume that a similar response accompanies the toxic effects of chemotherapy or radiotherapy in other sensitive organs, including liver and lungs.

All these pro-metastatic factors are released from the damaged tissues by different mechanisms. Accordingly, tissue damage-related hypoxia leads to synthesis and release of, for example, SDF-1, VEGF, and HGF/SF in an HIF-1α-dependent manner [67,83]. However, this process requires some time for these factors to be expressed through gene activation and transcription of mRNA and subsequent translation of mRNA into proteins. By contrast, alarmines or DAMPs (e.g., ATP, UTP, HMGB1, or S100 molecules) are released immediately from damaged and “leaky” cells [56,61,72].

Furthermore, it is important to highlight that chemotherapy- or radiotherapy-associated tissue and organ damage also activates developmentally early proteolytic cascades, such as the complement cascade (ComC), coagulation cascade (CoaC), and fibrynolytic cascade (FibC). It is well known that some of the activated proteolytic cleavage products of these cascades, such as C3a, C5a, thrombin, urokinase or uPAR, are directly or indirectly involved in cancer metastasis [84-88].

An important mechanism related to chemotherapy- or radiotherapy-mediated activation of CoaC and ComC is activation of blood platelets and release of platelet-derived microvesicles [84,89-91]. These small circular membrane fragments may transfer several platelet–endothelium cell adhesion receptors, for example, glycoprotein IIb/IIIa (CD41), Ib, IaIIa, and P-selectin (CD62P), to the surface of circulating tumor cells and thus facilitate attachment of CSCs or DTCs to the endothelium at the site of a future metastasis [89,92].

In addition to this pro-metastatic, adhesion-mediated effect, it has been reported that some cytostatics e.g., cyclophosphamide, by exerting direct toxicity to the endothelial wall, which affects the integrity of the endothelial barrier, may facilitate seeding of cancer cells into damaged organs through the disrupted endothelium [80].

**Figure 1:**
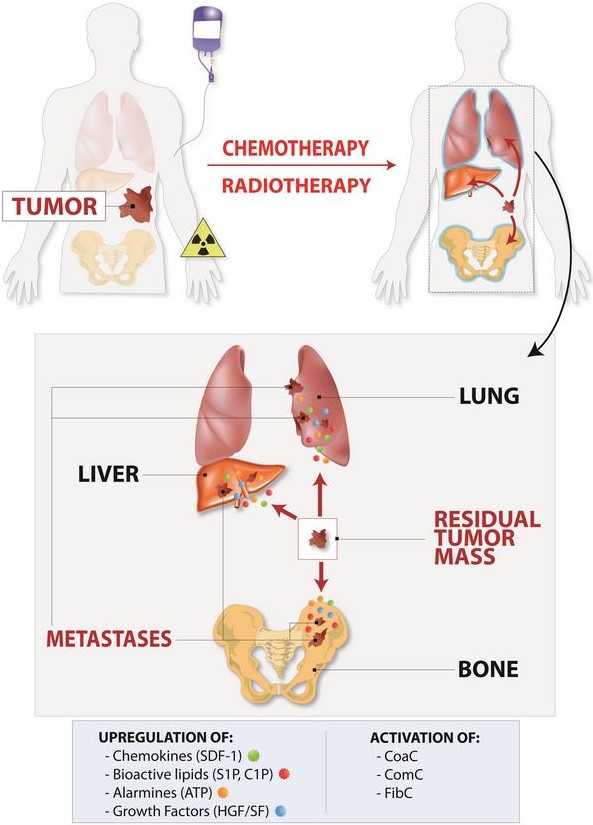
**Chemotherapy or radiotherapy induces a metastasis-receptive microenvironment in various organs.** One of the unwanted side effects of treatment is upregulation of several pro-metastatic and pro-survival factors, such as chemokines (e.g., SDF-1), growth factors (HGF/SF and VEGF), bioactive sphigophospholipids (S1P and C1P), and alarmines (ATP and UTP) in collateral-damaged tissues and organs. In parallel, in response to tissue damage, proteolytic cascades, such as the coagulation cascade (CoaC), complement cascade (ComC), and fibrynolytic cascade (FibC), are activated, which in different ways also enhance the metastasis of cancer cells that survived treatment. These most-resistant-to-therapy, and thus surviving, cancer cells are usually endowed with high endogenous motility. This mechanism plays an important role, primarily in metastasis of malignant cells to the tissues susceptible to the toxic effects of chemotherapy or radiotherapy, such as bones, lungs, liver and abdominal and pelvic cavities.

Figure 1 depicts our concept postulating creation of a chemotherapy- or radiotherapy-mediated pro-metastatic microenvironment in BM and other organs. This unwanted side effect is involved mainly in metastasis of tumor cells to tissues that are highly sensitive to damage by chemotheraputics as well as toxic doses of radiation (e.g., BM, liver, and lungs). Since BM tissue is highly sensitive to chemotherapy or radiotherapy, its damage facilitates creation of a metastasis-receptive microenvironment and is responsible for the occurrence of so frequent bone metastases as seen in the clinical setting.

### Practical clinical implications and the need for new therapeutic approaches

**Figure 2:**
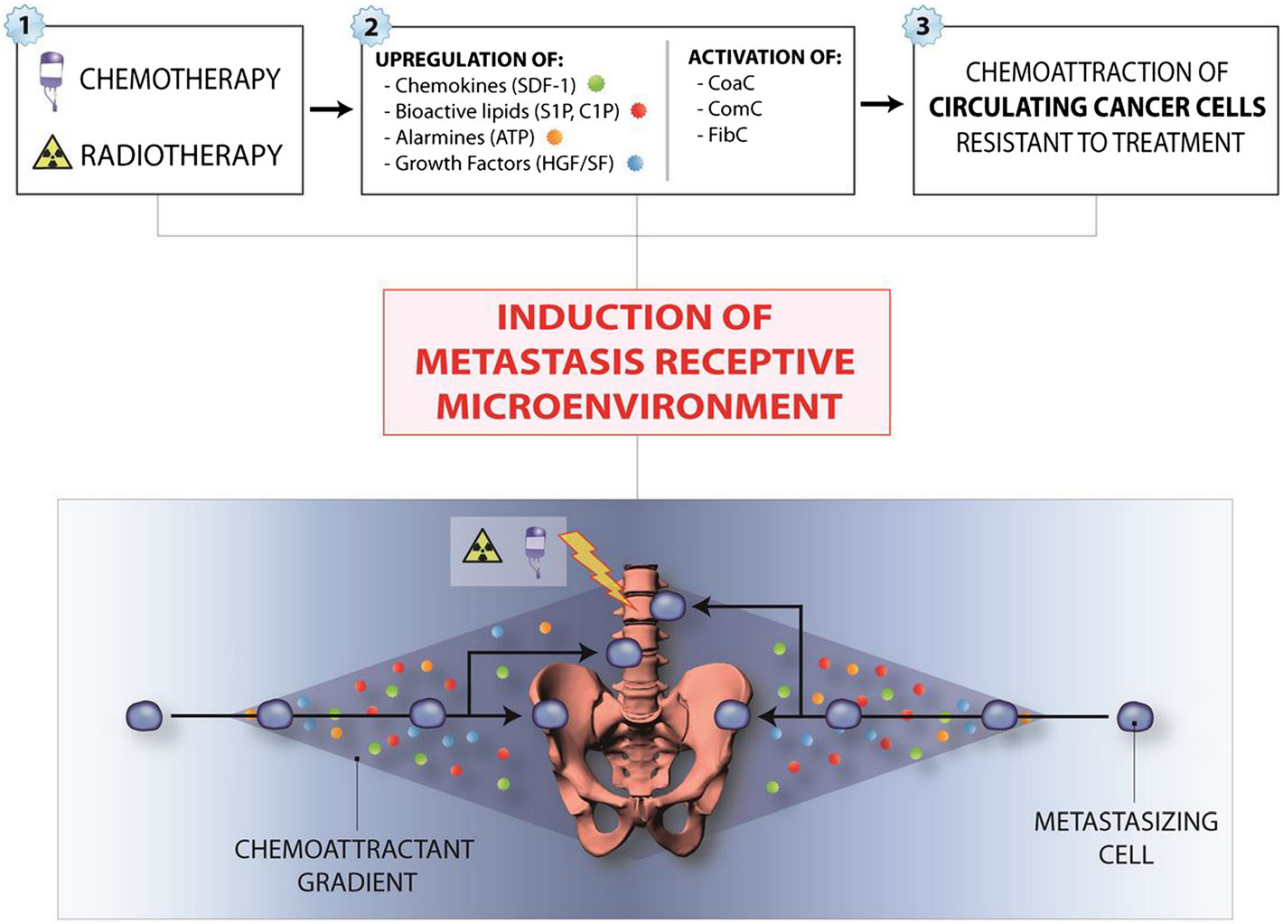
**Chemotherapy- or radiotherapy-mediated metastasis of cancer cells to bones.** Due to upregulation of several homing and pro-survival factors in response to BM microenvironment damage, circulating cancer cells may find a permissive environment in BM, which can originate growth of a bone metastasis. Similar mechanism may also operate in pelvic cavity after chemotherapy and lead to spread of ovarian cancer.

The natural propensity of CSCs or DTCs to migrate to distant metastasis-receptive locations and the fact that one of the unwanted side effects of chemotherapy or radiotherapy is induction of a metastasis-promoting microenvironment points to the important implication that standard treatment protocols should be followed by anti-metastatic strategies [61,93,94]. However, this is an obviously difficult task, because efficient anti-metastatic compounds are not currently available. One of the reasons for this unfortunate situation is that several developmental strategies for such drugs are based on targeting single chemoattractant-specific receptor axes (e.g., targeting SDF-1–CXCR4 or HGF/SF–c-Met). This is a somewhat questionable strategy, since tumor cells may respond simultaneously to several chemotactic factors and modulators of chemotactic responsiveness, and blockage of just one axis will be compensated by other unaffected pro-metastatic factors. The preferred sites of metastasis are tissues susceptible to damage by chemotherapy or radiotherapy such as bone marrow, and Figure 2 depicts a scenario where chemotherapy or radiotherapy induces a pro-metastatic microenvironment in bones for circulating CSCs or DTCs that survived treatment.

Nevertheless, in several well-controlled animal experimental models, it has been demonstrated that blockade of CXCR4 [50], CXCR7 [95], or c-Met [81] receptors by employing small molecular inhibitors; downregulation of these receptors by shRNA strategies [95]; *in vivo* administration of blocking antibodies against SDF-1 [50,61,96] or MCP-1 [80]; or application of S1P-binding aptamers [17] significantly diminished the process of chemotherapy- or radiotherapy-related dissemination of tumor cells to various organs.

However, the future of potent anti-metastatic drugs will depend on molecules that interfere with migration and adhesion processes of CSCs and DTCs downstream of surface receptors. It is obvious that the tumor specificity of such compounds will be a critical hurdle in development of such drugs. However, the simple and sad fact that metastases are responsible for 90% of cancer-associated mortalities points to the importance of developing such compounds for modern pharmacology.

### Implications for leukemia and lymphoma therapy

**Figure 3:**
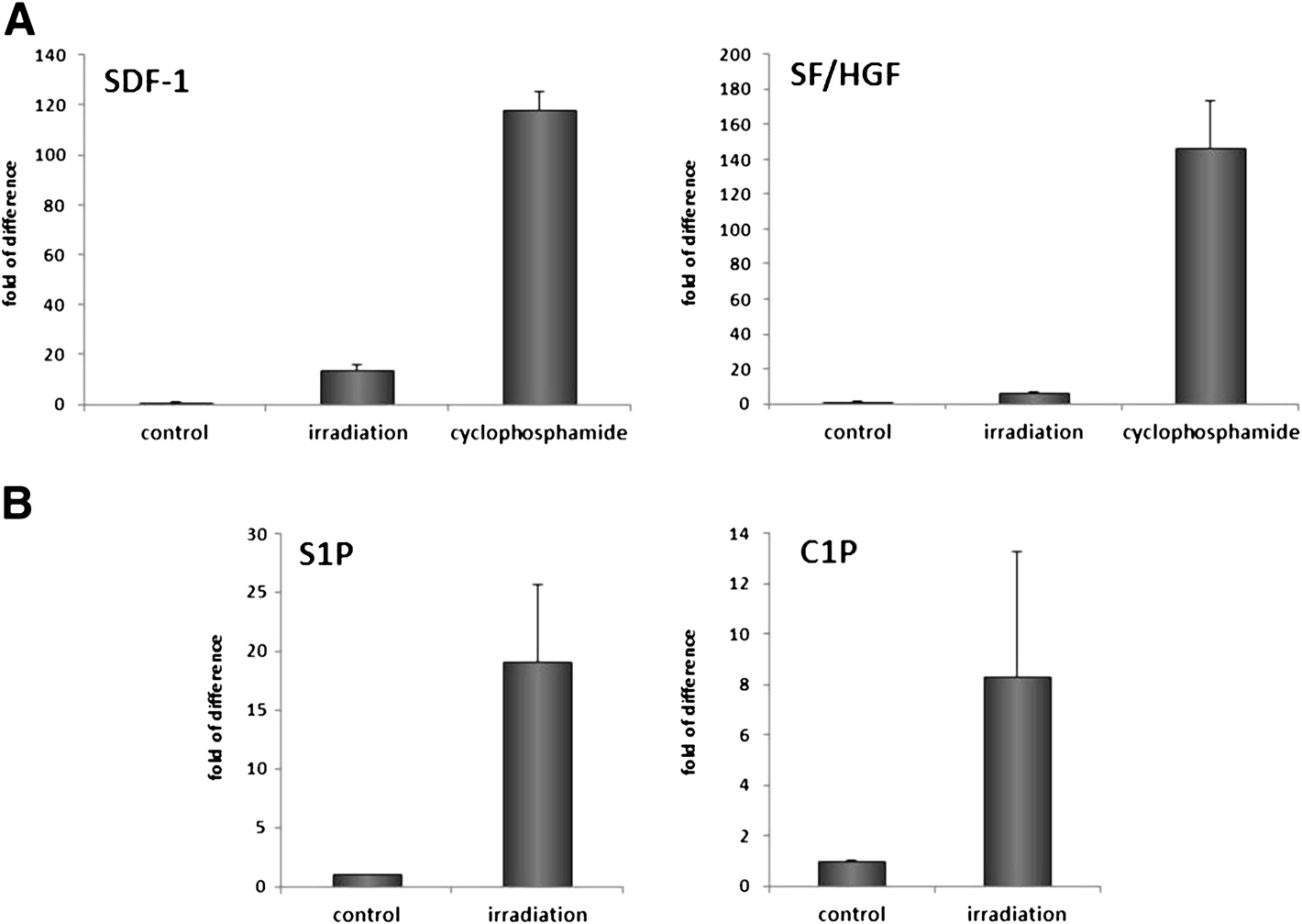
**Increase in expression of important chemoattractants and pro-survival factors in BM after *in vivo *exposure to γ-irradiation or cyclophosphamide treatment.** Panel **A**. Real-time PCR analysis of changes in the expression of mRNA for SDF-1 and SF in murine BM 24 hours after irradiation (750 cGy) or exposure to cyclophosphamide (200 mg/kg/bw/mouse). The experiment was repeated three times with four animals/group. Panel **B**. Mass spectrometry analysis shows that ceramide-1-phosphate (C1P) and sphingosine-1-phosphate (S1P) become upregulated in murine BM after conditioning for hematopoietic transplantation by lethal irradiation [17]. The data shown in panels A–B represent the combined results from three independent experiments carried out in triplicate per group.

However, while a major focus of this review is on solid tumors, which may metastasize and thus become unwanted inhabitants in BM or other organs, it is obvious that the same mechanisms may apply for malignant leukemia and lymphoma cells. As shown in Figure 3, both γ-irradiation as well as cyclophosphamide administration increase in BM expression of SDF-1, HGF/SF, S1P, and C1P, which on the one hand are potent chemotactic factors for tumor cells and, on the other hand, are potent pro-survival and anti-apoptotic agents for a variety of normal and malignant cells, including leukemia and lymphoma cells [3,17,80,81].

We propose that chemotherapy-induced BM toxicity may also have adverse effects on the most primitive leukemia or lymphoma stem cells. In response to treatment, the BM microenvironment releases several factors that may promote retention of malignant cells, and some of them involved in regeneration of marrow tissue may increase the survival of treatment-resistant cancer cells. To support this notion, it is well known that SDF-1, HGF/SF, S1P, and C1P are survival-increasing factors for many types of cells, including those from hematopoietic lineages [81,97].

Moreover, one of the treatment strategies in hematopoietic malignancies currently being explored is release of leukemia or lymphoma cells from the stem cell niches they occupy - by blocking the SDF-1–CXCR4 axis [24,98,99] or by blocking other homing signals to render them more sensitive to chemotherapeutic agents. Unfortunately, the upregulation of homing factors in BM as result of chemotherapy may increase expression of homing signals and thus retention of the most primitive and therapy-resistant cells in the BM microenvironment.

Leukemia and lymphoma CSCs and DTCs may also respond to homing cues in BM tissues of distant bones as well as migrate to organs where they find a permissive microenvironment (e.g., spleen, lymph nodes, and liver). This may, with time, promote spread of the malignancy.

### Implications for ovarian cancer metastasis and therapy

Ovarian cancer has the highest mortality rate among all the gynecologic tumors and in contrast to many other hematogenously metastasizing solid tumors disseminate within the peritoneal cavity [100]. It is often diagnosed at a late stage after tumor cells are already disseminated within pelvis. Thus, patients with ovarian cancer usually have at time of diagnosis locally advanced disease in the pelvic cavity, with continuous extension to uterus, fallopian tube, and the sigmoid colon. In contrast to prostate cancer, ovarian cancer in rare cases only metastasizes to BM or brain. It is also a chemotherapy sensitive malignancy. Therefore, radiotherapy is not a common option for its treatment.

Several genes, growth factors, adhesion molecules, chemokines, metalloproteinases and hormones have been reported to play critical role in progression and metastasis of ovarian cancer [100]. Many of them such as SDF-1, HGF/SF or S1P may be upregulated in tumor cells and microenvironment (e.g., ascites) in response to chemo/radiotherapy (Figure 1) [98-108].

As reported, SDF-1induces rapid intracellular calcium mobilization and activation of metalloproteinases MMP-2 and MMP-9 which enhance ERK1/2 signaling pathway leading to increase of cancer cell adhesion. SDF-1 also increases expression of CXCR4 at both the transcription and protein levels [102,106]. HGF/SF is another pro-metastatic factor that may be upregulated by chemo/radiotherapy, which after binding to tyrosine kinase receptor c-Met activates mitogenic pathways and thus plays an important role in tumor growth. Overexpression of HGF/SF or c-Met in ovarian cancer cells increases expression of α5 and β1 integrins, urokinase and metalloproteinases (MMP-2 and MMP-9) that have been shown to increase tumor growth and metastasis [103]. In addition HGF/SF activates also MAPKp42/44 and PI3K which leads to dissemination of ovarian cells [103,104]. In addition, HGF functions in morphological remodeling, invasion and migration of cancer cells, and decreases the expression of E-cadherin, β-catenin, and caveolin-1 at the cell membrane, mechanisms essential in promoting metastasis [106-108]. Finally, evidence accumulated that S1P signaling is also involved in epithelial ovarian tumorigenesis. This bioactive lipid is elevated in human ascites [105] and stimulates migration and invasion of ovarian epithelial cancer cells (EOC).

All this supports a concept that treatment of ovarian cancer should be from beginning combined with anti-metastatic approaches to lower risk of metastatic side effects of chemo/radiotherapy. It would also be interesting to investigate if so called metronomic chemotherapy, based in contrast to traditional chemotherapy on chronic, equally spaced administration of low doses of various chemotherapeutic drugs without extended rest periods that results in lower rate of metastasis/recurrence [109], is associated with reduced release of pro-metastatic factors from damaged organs.

## Conclusions

We propose a novel concept whereby the unwanted and underappreciated side effects of chemotherapy or radiotherapy create a metastasis-receptive microenvironment in bones as well as in other organs of the body (e.g., pelvic cavity). Upregulation of several chemokines, growth factors, alarmines, bioactive phosphosphingolipids as well as activation of CoaC, ComS, FibC and metalloproteinases, *in toto,* enhances and modulates trafficking and survival of CSCs and DTCs that survived treatment. This implies a challenge for modern pharmacology to develop powerful anti-metastatic compounds that can be combined with standard chemotherapy and radiotherapy protocols or applied as a follow up to these protocols.

## Competing interests

The authors declare that they have no competing interests.

## Authors’ contributions

MZR - conceived idea and wrote paper SSK - reviewed and approved manuscript MK - referenced mansucript TJ - prepared Figures 1 and 2 GS - approved and approved manuscript, prepared Figure 3. All authors read and approved the final manuscript.
